# Assessing the job preferences of public health specialist doctors: a discrete choice experiment

**DOI:** 10.1017/S1463423626101510

**Published:** 2026-07-28

**Authors:** Buşra Tozduman, İrem Zengi

**Affiliations:** 1 Dokuz Eylul University, Faculty of Medicine, Department of Public Health, Epidemiology Subsection, Izmir, Türkiye; 2 Ministry of Health of the Republic of Türkiye, General Directorate of Public Health, Ankara, Türkiye

**Keywords:** discrete choice experiment, health workforce, job preferences, public health specialist, willingness to pay

## Abstract

**Background:**

Public health specialists (PHSs) are responsible for developing and implementing health promotion policies and can contribute to healthcare delivery at all levels. Discrete choice experiments (DCEs) can inform policy by identifying healthcare professionals’ job preferences and the trade-offs they are willing to make between job attributes. This study aimed to investigate job preferences among PHSs, including trade-offs between job attributes and the probability of choosing specific job scenarios.

**Methods:**

This cross-sectional analytical study was conducted among 183 PHSs. Job attributes and levels were determined through a literature review and semistructured interviews with specialists. The questionnaire was administered online. A mixed logit model was used to estimate preferences for different attribute levels. Latent class analysis (LCA) was performed to identify subgroups with similar preference patterns. Simulations of job uptake probabilities were also conducted and willingness-to-pay (WTP) estimates were calculated.

**Results:**

Five of the six job attributes significantly influenced preferences, ranked as follows: proximity to family/friends, working environment, salary, developmental status of the workplace, and managerial position. LCA indicated that preference weights varied across subgroups defined by sociodemographic and professional characteristics.

**Conclusions:**

In addition to monetary incentives, ensuring proximity to family and providing a supportive working environment appear to be the most effective strategies for improving the employment of PHSs. Financial incentives are particularly important for managerial roles, especially among junior PHSs without prior managerial experience.

## Background

The density of the health workforce is directly related to the scope of health services and key health outcomes, such as maternal, infant and under-five child mortality rates (Robinson and Wharrad, 2001; Anand and Bärnighausen, 2004; Chen *et al*., 2004). Nevertheless, many countries face challenges in training, recruiting, and deploying their health workforce (Araújo and Maeda, 2013). Among OECD (Organisation for Economic Co-operation and Development) countries, Türkiye has the lowest number of physicians per 100,000 population (‘Health resources - Doctors - OECD Data’, n.d.). In addition, there is a marked geographical imbalance in workforce distribution. Despite policies such as increased physician training, compulsory service, and financial incentives, these challenges persist (Yildirim *et al*., 2020). Physician migration further exacerbates the problem. Over the past decade, the number of physicians obtaining a ‘Certificate of Good Standing’ to work abroad has increased markedly, rising from 59 to over 3,000 annually (‘X, Turkish Medical Association’, 2023; ‘Artı Gerçek’, 2024). A recent multicenter study reported that 71% of final-year medical students are definitely or potentially planning to work abroad (Eser *et al*., 2023).

In Türkiye, public health specialization is a four-year residency programme. Public health specialists (PHSs) are trained to assess population health, develop and implement health policies and manage health services. However, only 15.6% hold managerial positions, and many are assigned to low-population, remote areas rather than to central institutions responsible for designing and coordinating health programmes (Report on the Job Description and Employment of Public Health Specialists Online Workshop, 2021; Report on the Employment and Managerial Status of Public Health Specialists in the Ministry of Health Positions, 2022). These structural challenges highlight the need for more effective employment and retention policies for PHSs.

Discrete choice experiments (DCEs) are quantitative methods based on the assumption that goods and services can be described by their attributes and that the value of these goods derives from the combination of those attributes (Ryan *et al*., 2001). DCEs enable the identification of healthcare workers’ job preferences by quantifying the relative importance of different attributes, the trade-offs individuals are willing to make, and how variations in these attributes influence job choices (WHO, 2012).

Building on this approach, the aims of this study were to examine (a) job preferences and associated individual characteristics, (b) the salary PHSs are willing to forgo for preferred working conditions, and (c) the impact of changes in job characteristics on the probability of choosing one job over another.

## Methods

This was a cross-sectional analytical study. The target population consisted of PHS doctors actively working in the field (*n* = 531) (Report on the Employment and Managerial Status of Public Health Specialists in the Ministry of Health Positions, 2022).

### Survey design

To identify relevant job characteristics (attributes) and their levels, a literature review was conducted (İşlek and Şahin, 2023; Cheng *et al*., 2024; Luo *et al*., 2024; Tozduman and Sözmen, 2024). These were further refined through semi-structured individual consultations with eight PHSs, focusing on the relevance, clarity, and contextual appropriateness of the attributes and levels. The final set comprised six attributes, each with two to four levels (Table 1).

**Table 1.** Job attributes, levels, and definitions Table 1 long description.

Attributes	Levels	Definition
Developmental status of current workplace	1–2	Most developed
	3–4	Moderately developed
	5–6	Least developed (standard of living, educational level, socioeconomic status)
Facility	Provincial health directorate	
	County health directorate/Community health centre	
Manager position	Yes	
	No	
Salary	60.000 TL (7 792 $)*	Total monthly income from working in the facility
	66.000 TL (8 571 $)*	
	72.000 TL (9 350 $)*	
	78.000 TL (10 129 $)*	
Working environment	Ideal	Good communication between other HCWs and managers, HCWs highly motivated for work
	Not ideal	Poor communication between other HCWs and managers, HCWs are not highly motivated for work
Proximity to family/friends	Near	<90 km
	Far	>90 km

*Note:* *converted with purchasing power parity (PPP) rate(‘World Bank Open Data’, n.d.).

We used JMP Pro 14 (SAS Institute, Cary, NC) to generate the choice sets. Each questionnaire consisted of 10 choice sets, comprising a total of 20 job profiles with different combinations of attribute levels. Six versions of the experimental design were created to improve statistical efficiency.

To reduce cognitive burden, a partial profile design was employed, ensuring that no more than four attributes varied within each choice set. The design was assessed for orthogonality and balance in the distribution of attribute levels.

The DCE instrument also included questions on respondents’ sociodemographic characteristics and professional experiences. Data were collected between September and November 2023. Participants received one version of the questionnaire online and were asked to choose between two job alternatives in each choice set.

To identify respondents whose choices violated basic rationality assumptions, a rationality (dominance) test was incorporated as an additional choice set. In this set, all attributes were held constant except for salary and working environment; the alternative with a higher salary and an ideal working environment was expected to be preferred. Responses to this rationality test were not included in the main analysis.

### Respondents

The minimum sample size was estimated as 100 using Johnson and Orme’s method (Orme, 1998; Johnson and Orme, 2003). However, rather than sampling, we aimed to reach the entire target population of PHSs.

### Data analysis

#### Discrete choice experiment

The analysis of DCE data is based on the random utility model. Within this framework, an individual *n* is assumed to choose among alternative job options by selecting the one that provides the highest utility. The utility (*U*) derived from a given job is composed of two components: the deterministic and the random components. The utility, *U*, that individual *n* derives from job *i* is specified as:

U_ni_ = V_ni_ + ϵ_ni_ V_ni_ = α_1_ + β_1_x_1ni_ + β_2_x_2ni_ + … + β_m_x_mni_

Here V_ni_ represents the deterministic component specified as a function of m job attributes (x_1_, x_2_, ….. x_m_), with ß coefficients capturing the strength of preference for each attribute level. The random component, ϵ_ni_, captures unobserved factors and individual-level preference heterogeneity (Vujicic *et al*., 2010; WHO, 2012).

All DCE data were entered into Microsoft Excel 2016 (Microsoft Corporation, USA). Descriptive statistics are presented as means (standard deviations) or frequencies and percentages. Salary was specified as a continuous variable, while all other attributes were dummy coded (1 indicating presence and 0 indicating absence).

A mixed logit (MXL) model was used to estimate preferences for attribute levels, implemented in Stata® 15.0 (Stata Corporation, USA) using user-written commands (Hole, 2013). The salary attribute was specified as fixed to enable the estimation of willingness-to-pay (WTP), whereas all other attributes were treated as random parameters. WTP estimates were calculated as the ratio of the coefficient of each attribute level to the negative of the salary coefficient. In addition, changes in predicted choice probabilities resulting from variations in attribute levels were estimated using Hole’s mixlpred command (Hole, 2013).

#### Latent class analysis (LCA)

Given the presence of significant preference heterogeneity, LCA was conducted to identify subgroups of respondents with similar preference patterns, using the poLCA package in R (version 4.3.1). Sociodemographic and professional characteristics (gender, marital status, having children, hometown, income, years of experience as a PHS, work experience in moderately or least developed regions, and managerial experience) were included to explore differences across latent classes.

The optimal number of classes was determined based on model fit criteria, including the Akaike Information Criterion (AIC) and Bayesian Information Criterion (BIC), as well as the interpretability of the resulting class structure. The model estimated class membership probabilities for each respondent, and individuals were assigned to the class for which they had the highest posterior probability.

## Results

Of the 531 PHSs, 183 (34.4%) completed the questionnaire. Four respondents (2.1%) failed the rationality test; however, model estimates with and without these respondents did not differ substantially, and they were therefore retained in the main analysis. No participants exhibited dominant preferences, indicating that all respondents traded off between attribute levels.

The characteristics of the respondents are presented in Table 2. Overall, 69.9% were female, and the median age was 36.2 years. Most respondents were married (74.9%), and 57.9% had children. Only 6.6% reported a rural background (i.e., having grown up in a village). Regarding income, 12.6% reported that their income was lower than their expenditure. Nearly half of the participants were working in a county health directorate or in a developed region.

**Table 2.** The general characteristics of public health specialists Table 2 long description.

	Mean±S.S.
Age	36.2±5.5
	**n (%)**
Gender (female)	128 (69.9%)
Marital status (married)	137 (74.9%)
Have child (yes)	106 (57.9%)
Hometown	
City	74 (40.4%)
County	97 (53.0%)
Village	12 (6.6%)
Income	
Less than expenditure	23 (12.6%)
Equal to expenditure	85 (46.4%)
More than expenditure	75 (41.0%)
Facility;	
Health directorate	6 (3.3%)
Provincial health directorate	59 (32.2%)
County health directorate	93 (50.8%)
Community health centre	18 (9.8%)
Public health laboratory/Hospital	7 (3.8%)
Working years as PHS	4.0±3.7
Developmental status of current workplace	
1-2 (most developed)	102 (55.8%)
3-4 (moderately developed)	42 (23.0%)
5-6 (least developed)	39 (21.4%)
Performing compulsory service	56 (30.6%)
Completed compulsory service	127 (69.4%)
Manager position currently (yes)	28 (15.3%)
Manager position before (yes)	51 (27.9%)

A total of 15.2% currently held managerial positions, while 27.9% had prior managerial experience. The sample broadly reflected the overall distribution of PHSs in terms of facility type and managerial roles (Report on the Employment and Managerial Status of Public Health Specialists in the Ministry of Health Positions, 2022) (Table 2). More than half of the respondents were working in highly developed regions. In addition, 30.6% were still completing compulsory service, whereas 69.4% had already completed it.

Table 3 presents the MXL model estimates. The coefficients were in the expected direction, with positive signs indicating increased utility relative to the reference levels.

**Table 3.** Estimation results from the mixed logit model Table 3 long description.

	Mixed logit model	
	β (S.E.)	β std. (S.E.)
**Salary**	10^−4^ (10^−5^)***	–
**Developmental status (Base:Least)**		
**Most**	1.27 (0.19)***	1.16 (0.24)***
**Moderately**	0.84 (0.16)***	0.08 (0.40)
**Facility (Base: County health directorate/Community health centre)**	0.05 (0.16)	1.40 (0.24)***
**Manager position (base:yes)**	0.69 (0.18)***	1.39 (0.24)***
**Working environment (base:not ideal)**	2.68 (0.28)***	2.0 (0.32)***
**Proximity to family/friends (base: far)**	2.27 (0.23)***	1.58 (0.24)***
**Observations**	3660	
**Log likelihood (null)/(model)**	−830.2/−758.3	
**LR χ^2^, Prob > chi2**	143.74 <0.001	
**AIC/BIC**	1542.6/1623.3	
**McFadden’s R^2^**	0.086	

*Note:* **p < 0.01, ***p < 0.001; AIC: Akaike information criterion; BIC: Bayesian information criterion.

The results showed that PHSs preferred jobs located in the most and moderately developed regions compared with those in the least developed regions (*β* (S.E. ) = 1.27 (0.19); *p* < 0.001, *β* (S.E.) = 0.84 (0.16); *p* < 0.001, respectively). Higher salary was also positively associated with job choice (*β* (S.E.) = 10^−4^ (10^−5^) per Turkish lira (TL); *p* < 0.001), along with an ideal working environment (*β* (S.E.) = 2.68 (0.28); *p* < 0.001) and proximity to family/friends (*β* (S.E.) = 2.27 (0.23); *p* < 0.001). Holding a managerial position was associated with a lower likelihood of selecting a job (*β* (S.E.) = 0.69 (0.18); *p* < 0.001). The MXL results also indicated significant unobserved preference heterogeneity across respondents, as evidenced by statistically significant standard deviations of the random parameters.

According to the LCA results, three distinct classes of participants were identified (Table S2). Class 1 (32%) comprised predominantly male respondents, more likely to originate from counties or villages, with income equal to or lower than expenditure, and with longer work experience and prior managerial experience.

Class 2 (35%) consisted mainly of female respondents, with limited work experience in moderately or least developed regions and no managerial experience.

Class 3 (33%) included mostly single participants without children, originating from urban areas, with higher income, fewer years of work experience, and minimal managerial experience.

**Figure 1.**
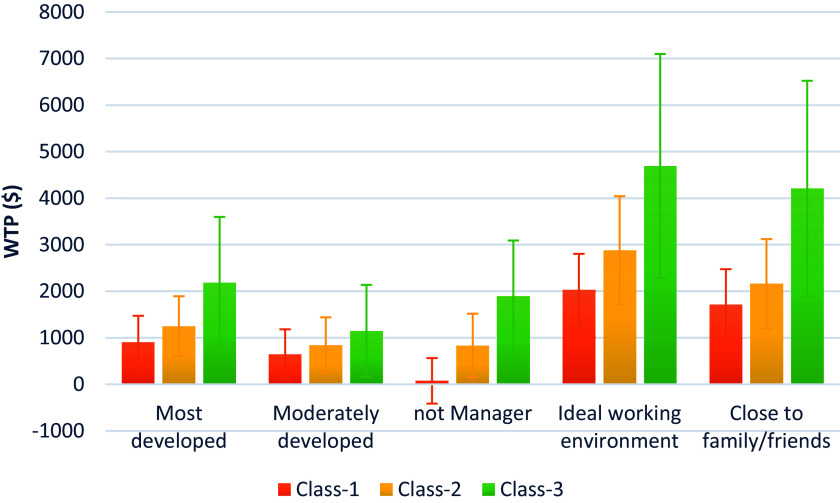
Willingness to pay estimation for three latent class groups.

Figure 1 presents the WTP estimates for the three latent classes, converted using purchasing power parity (PPP), along with 95% confidence intervals (CIs) (the exchange rate from October 2023 of 1 US dollar = 27.8 TL, 1 Euro = 29.3 TL, PPP rate = 7.7) (‘TCMB Central Bank of the Republic of Turkey Currency Exchange Rates’, n.d.; ‘World Bank Open Data’, n.d.). Across all classes, the relative ranking of attributes was consistent. Participants in Class 1 exhibited lower WTP estimates for all attributes and did not value managerial positions. In contrast, participants in Class 2 showed higher WTP estimates across attributes, while the highest WTP values were observed in Class 3.

**Figure 2.**
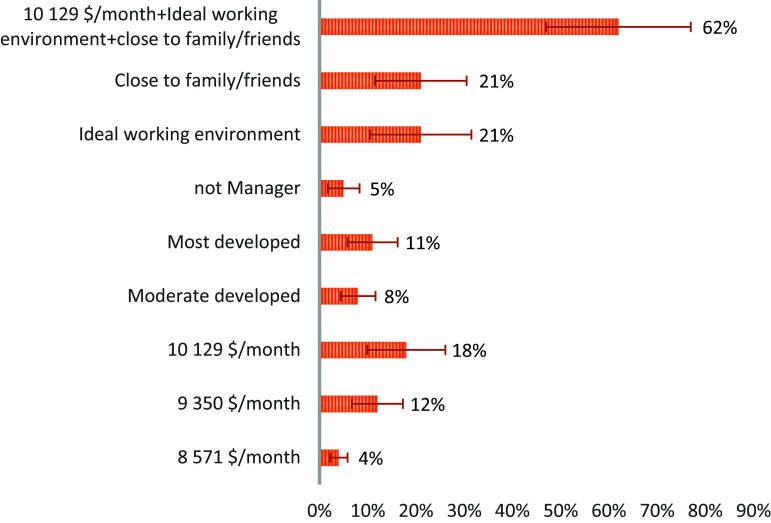
Changing in probabilities of taking a job with changing job condition.

Figure 2 illustrates the predicted probabilities of job uptake under varying job conditions, along with their standard deviations. Holding all other attributes constant (ceteris paribus), increasing the salary from 7 792 $ to 8 571 $, is associated with a 4% increase in the probability of choosing that job.

Jobs located in moderately and most developed regions are associated with 8% and 11% higher probabilities of selection, respectively, compared with those in the least developed region. In contrast, a managerial role is associated with a 5% decrease in the probability of job choice.

An ideal working environment and proximity to family and friends each increase the probability of choosing a job by 21%. The model further predicts that a combination of proximity to family/friends, an ideal working environment, and a monthly salary of 10 129 $ would increase the likelihood of job selection by 62%.

Additional examples of combined job attribute improvements (incentive packages) and their effects on job choice probabilities are presented in Supplementary Table 3.

## Discussion

This study used a DCE to assess job preferences among PHSs. Five of the six job attributes were found to significantly influence choice behaviour. Overall, participants preferred positions located in more developed regions, close to family and friends, with higher salaries, non-managerial roles, and favourable working conditions.

The MXL model revealed substantial preference heterogeneity across all attributes, indicating variability in how individuals value different job characteristics. This finding was further supported by the LCA, which demonstrated that preferences varied according to respondents’ sociodemographic and professional characteristics.

This study identified working environment and proximity to family and friends as the most influential determinants of job preferences. This finding is consistent with previous research, including a study among Chinese healthcare administration students that also emphasized the importance of working conditions (S. Liu *et al*., 2019). Poor working environments have been shown to increase pressure on physicians and contribute to burnout (Azam *et al*., 2017) underscoring the importance of organizational factors in workforce retention. In line with this, a 2017 survey by the Turkish Ministry of Health reported that nearly half of healthcare staff believed that community health centres could function more effectively if roles and responsibilities were more clearly defined (Healthcare Employee Satisfaction Survey Turkey, 2017). In primary care settings, where multidisciplinary teams – including PHSs, family physicians, midwives, nurses, medical secretaries, environmental health technicians, and health officers – work together, effective teamwork is critical. Such collaboration depends on clear role definitions, motivated staff, and supportive relationships between supervisors and team members.

Proximity to family and friends was also a significant determinant of job preferences, consistent with findings from studies conducted in Australia and Canada (Szafran *et al*., 2001; Matthews *et al*., 2012; Harding *et al*., 2016). Social support is a well-established factor in physician well-being, with lack of support associated with increased risks of depression and burnout(Kuhn and Flanagan, 2016).

In Türkiye, regulations exist to facilitate family reunification; however, physicians are classified as strategic personnel and are subject to certain restrictions. These policies primarily apply to spouses, while reunification with other family members is permitted only under specific health-related circumstances.

Notably, in this study, single participants valued proximity to family and friends to a similar extent as married PHSs, suggesting that social support extends beyond traditional family structures and plays a broader role in job satisfaction and retention.

Consistent with previous studies, our findings confirm that salary is a key determinant of job preferences (Vujicic *et al.*, 2010; Liu *et al.*, 2019; Liu *et al.*, 2019; Karyani *et al*., 2020; Bao *et al*., 2023). This is also reflected in national data: a survey conducted by the Turkish Ministry of Health identified remuneration as the most influential factor in job satisfaction and one of the most frequently cited areas requiring reform (*Healthcare Employee Satisfaction Survey Turkey*, 2017). Similarly, a DCE conducted among general practitioners in Türkiye ranked salary as the second most important job attribute, further underscoring its role in recruitment and retention strategies (İşlek and Şahin, 2023).

Participants also placed significant value on the developmental status of the workplace, consistent with previous studies showing that healthcare workers tend to prefer centrally located positions (Kolstad, 2011; Smitz *et al*., 2016; Liu *et al*., 2018; İşlek and Şahin, 2023). Rural and remote areas are generally considered less attractive due to limited educational opportunities for children, inadequate infrastructure, and fewer employment options for spouses (Lehmann *et al*., 2008; Liu *et al.*, 2018).

Although certain policies aim to mitigate these disparities – such as differentiated compulsory service durations and supplementary payments based on development status – these measures appear insufficient to address workforce shortages in underdeveloped regions (Basic Health Services Law, 1987). While rural upbringing has been associated with an increased likelihood of returning to rural practice (Lehmann *et al.*, 2008), our findings did not support this pattern. PHSs evaluated the developmental status of workplaces similarly, regardless of rural or urban background. Notably, WTP estimates were highest among participants predominantly from urban areas, suggesting stronger preferences for favourable working conditions within this group.

Managerial position also had a significant effect on job preferences. In Türkiye, contractual health management was introduced in 2011 as part of the Health Transformation Program to improve efficiency, accountability, and sustainability in healthcare management (Armağan Kaygusuz, 2022) (Law Decree Regarding The Organızation And Duties Of The Ministry Of Health And Affiliated Institutions, 2011). However, previous studies have shown that healthcare managers often report limited authority, insufficient autonomy, and unclear role definitions (Topcu, 2018).

Participation in decision-making processes is associated with higher job satisfaction (Whalley *et al*., 2006). Consistent with this, a 2010 survey by the Ministry of Health reported higher satisfaction among administrative personnel compared with clinical staff (Healthcare Employee Satisfaction Survey Turkey, 2010) and a subsequent survey in 2017 indicated that managerial roles may enhance satisfaction through financial incentives, promotion opportunities, and increased responsibility (Healthcare Employee Satisfaction Survey Turkey, 2017).

In addition, factors such as experience, autonomy, resilience to burnout, and occupational prestige have been shown to influence job satisfaction (Visser *et al*., 2003). In the present study, holding a managerial position did not have a negative effect on job preferences among PHSs with more than five years of experience or prior managerial roles. This may suggest that, with increasing experience, greater adaptability and adjusted expectations contribute to higher levels of job satisfaction.

## Conclusions

Key job features associated with improved recruitment and retention include proximity to family and a supportive working environment characterized by strong communication and high motivation within multidisciplinary healthcare teams. Financial incentives also play a significant role, particularly for managerial positions, where additional compensation appears to be especially important for junior PHSs without prior managerial experience.

Overall, combinations of monetary and non-monetary incentives tailored to individual characteristics are likely to be more effective. Consistent with previous DCE evidence, policies that account for preference heterogeneity – rather than adopting uniform, one-size-fits-all approaches – are more likely to enhance the recruitment and retention of PHSs.

## Strengths and limitations

This study is, to our knowledge, the first to apply a DCE methodology to investigate job preferences among PHSs in Türkiye. A further strength is the inclusion of a rationality (dominance) test within the choice sets, enabling the identification of responses that violate basic rationality assumptions.

Several limitations should be considered. The response rate of 34.4% may be regarded as a limitation; however, it is comparable to those typically reported in web-based surveys. To maximize participation and approximate comprehensive coverage of the target population, the questionnaire was disseminated repeatedly through multiple online channels. Despite these efforts, the possibility of non-response bias remains, and the findings should therefore be interpreted with caution in terms of their generalizability.

In addition, the hypothetical nature of DCEs may result in discrepancies between stated and actual (revealed) preferences. Furthermore, DCEs necessarily simplify complex decision-making contexts by limiting the number of attributes and levels; as a result, other relevant job characteristics influencing employment decisions may not have been captured. Therefore, policy makers should interpret and validate DCE-derived evidence in conjunction with real-world data before implementing specific intervention packages (Araújo and Maeda, 2013).

## Supporting information

10.1017/S1463423626101510.sm001 Tozduman and Zengi supplementary material Tozduman and Zengi supplementary material

## Data Availability

The data supporting the findings of this study are not publicly available but can be made available upon reasonable request to the corresponding author.

## Supplementary material

The supplementary material for this article can be found at https://doi.org/10.1017/S1463423626101510.

## Table 1. Long description

A table with six attributes, each with two to four levels, detailing job characteristics for health workers. The table has three columns: Attributes, Levels, and Definition. The attributes include Developmental status of current workplace, Facility, Manager position, Salary, Working environment, and Proximity to family/friends. The Levels column lists the different levels for each attribute, and the Definition column provides descriptions for each level. The table is structured to show the relevance, clarity, and contextual appropriateness of the attributes and levels for health workers.

Navigate back to Table 1..

## Table 2. Long description

A table summarizing characteristics of public health specialists. The table has 16 rows and 3 columns. The columns are labeled 'Mean±S.S.', 'n (%)', and various characteristics. Row 1: Age, 36.2±5.5. Row 2: Gender (female), 128 (69.9 percent). Row 3: Marital status (married), 137 (74.9 percent). Row 4: Have child (yes), 106 (57.9 percent). Row 5: Hometown (City), 74 (40.4 percent). Row 6: Hometown (County), 97 (53.0 percent). Row 7: Hometown (Village), 12 (6.6 percent). Row 8: Income (Less than expenditure), 23 (12.6 percent). Row 9: Income (Equal to expenditure), 85 (46.4 percent). Row 10: Income (More than expenditure), 75 (41.0 percent). Row 11: Facility (Health directorate), 6 (3.3 percent). Row 12: Facility (Provincial health directorate), 59 (32.2 percent). Row 13: Facility (County health directorate), 93 (50.8 percent). Row 14: Facility (Community health centre), 18 (9.8 percent). Row 15: Facility (Public health laboratory/Hospital), 7 (3.8 percent). Row 16: Working years as PHS, 4.0±3.7.

Navigate back to Table 2..

## Table 3. Long description

A table with estimation results from a mixed logit model. The table has 12 rows and 3 columns. The columns are labeled as Salary, Developmental status (Base:Least), Facility (Base: County health directorate/Community health centre), Manager position (base: yes), Working environment (base:not ideal), Proximity to family/friends (base: far), Observations, Log likelihood (null)/(model), LR χ2, Prob > chi2, AIC/BIC, McFadden's R2. The rows list different variables and their corresponding coefficients (β) and standard errors (S.E.) for the mixed logit model. The table includes specific values for each variable, indicating their impact on the model. Notable trends include positive coefficients for variables like 'Most' and 'Moderately' under Developmental status, and significant values for Manager position and Working environment.

Navigate back to Table 3..
