# Supplementary material for: Assessing the job preferences of public health specialist doctors: a discrete choice experiment
Source: Prim Health Care Res Dev. 2026 Jul 28;27:e78. doi: 10.1017/S1463423626101510 (PMC13439023; doi:10.1017/S1463423626101510)
Supplement: Supplementary file 1 [file S1463423626101510sup001.docx]

# Criteria for choosing the number of classes in the latent class analysis

Some participant characteristics (gender, marital status, having a child, hometown, income, years as a PHS, work experience in moderately or least developed regions, and managerial experience) were used to categorize respondents into groups with similar characteristics. The following R commands were used for the analysis:

f <- cbind(c1, c2, c3, c4, c5, c6, c7, c8)~1

lc <- poLCA(f, demograf, nclass=3, maxiter=1000, graphs=TRUE, tol=1e-10, na.rm=TRUE, probs.start=NULL, nrep=1, verbose=TRUE, calc.se=TRUE)

The analysis was conducted with **nclass = 2, 3, and 4**, and model selection was based on AIC and BIC values:

- **Two-class model:** AIC = 1793.855, BIC = 1848.41
- **Three-class model:** AIC = 1780.456, BIC = 1863.903
- **Four-class model:** AIC = 1783.682, BIC = 1896.014

For practical reasons, the **three-class model** was preferred. The table below presents the probabilities of each variable within the three classes.

Table S1. Conditional item response probabilities by outcome variable for each class

|  | Class 1 | Class 2 | Class 3 |
| --- | --- | --- | --- |
| Gender (Male) | 0,40 | 0,13 | 0,36 |
| Marital status (single) | 0,02 | 0 | 0,74 |
| Have child (no) | 0,10 | 0,17 | 1,0 |
| Hometown (city) | 0,28 | 0,39 | 0,53 |
| Income (more than expenditure) | 0,33 | 0,37 | 0,51 |
| Working year as PHS (<5 years) | 0,46 | 0,69 | 0,83 |
| Work experience in moderately or least developed regions (no) | 0,14 | 0,64 | 0,21 |
| Managerial experience (no) | 0,26 | 1,0 | 0,70 |

**lc$predclass** command was used to predict class memberships, which assigns respondents to the most probable latent class based on their posterior probabilities.

Table S2. General characteristics of the three latent class groups

|  | | **Class 1**  **(n=58)** | **Class 2**  **(n=65)** | **Class 3**  **(n=60)** |
| --- | --- | --- | --- | --- |
| Gender  Female  Male | 32 (25,0%)  26 (47,3%) | | 59 (46,1%)  6 (10,9%) | 37 (28,9%)  23 (41,8%) |
| Marital status  Married  Single | 57 (41,6%)  1 (2,2%) | | 65 (47,4%)  0 | 15 (10,9%)  45 (97,8%) |
| Have child  Yes  No | 51 (48,1%)  7 (9,1%) | | 55 (51,9%)  10 (13%) | 0  60 (77,9%) |
| Hometown  City  County/Village | 15 (20,3%)  43 (39,4%) | | 26 (35,1%)  39 (35,8%) | 33 (44,6%)  27 (24,8%) |
| Income  Less than/equal to expenditure  More than expenditure | 39 (36,1%)  19 (25,3%) | | 40 (37,0%)  25 (33,3%) | 29 (26,9%)  31 (41,3%) |
| Working year as PHS  <5 years  ≥5 years | 24 (19,8%)  34 (54,8%) | | 46 (38,0%)  19 (30,6%) | 51 (42,1%)  9 (14,5%) |
| Work experience in moderately or least developed regions  Yes  No | 52 (42,6%)  6 (9,8%) | | 22 (18,0%)  43 (70,5%) | 48 (39,3%)  12 (19,7%) |
| Managerial experience  Yes  No | 47 (74,6%)  11 (9,2%) | | 0  65 (54,2%) | 16 (25,4%)  44 (36,7%) |

Table S3. Probabilities of taking a job with changing job conditions

|  | Salary | Developmental status | Manager position | Working environment | Proximity to family/ friends | increase in the probability of being selected |
| --- | --- | --- | --- | --- | --- | --- |
| Base | 60 000 | Least | Yes | Not ideal | Far |  |
| Package 1 | 66 000 | Most | Yes | Ideal | Near | 0,62±0,15 |
| Package 2 | 72 000 | Moderately | Yes | Ideal | Near | 0,67±0,14 |
| Package 3 | 78 000 | Least | No | Not ideal | Far | 0,29±0,13 |
| Package 4 | 66 000 | Most | No | Ideal | Near | 0,67±0,14 |
| Package 5 | 72 000 | Least | No | Not Ideal | Near | 0,44±0,15 |
| Package 6 | 78 000 | Moderately | No | Ideal | Near | 0,76±0,12 |
| Package 7 | 66 000 | Moderately | No | Ideal | Far | 0,45±0,16 |
| Package 8 | 72 000 | Most | Yes | Not Ideal | Far | 0,28±0,13 |
| Package 9 | 78 000 | Least | No | Ideal | Far | 0,54±0,16 |
| Package 10 | 66 000 | Moderately | Yes | Ideal | Near | 0,60±0,16 |
| Package 11 | 72 000 | Least | Yes | Ideal | Near | 0,61±0,15 |
| Package 12 | 78 000 | Most | Yes | Not ideal | Far | 0,37±0,15 |
